# Regulation of hBD-2, hBD-3, hCAP18/LL37, and Proinflammatory Cytokine Secretion by Human Milk Oligosaccharides in an Organotypic Oral Mucosal Model

**DOI:** 10.3390/pathogens10060739

**Published:** 2021-06-11

**Authors:** Ulvi K. Gürsoy, Krista Salli, Eva Söderling, Mervi Gürsoy, Johanna Hirvonen, Arthur C. Ouwehand

**Affiliations:** 1 Department of Periodontology, Institute of Dentistry, University of Turku, Lemminkäisenkatu, 2, 20520 Turku, Finland; ulvgur@utu.fi (U.K.G.); esoder@utu.fi (E.S.); mervi.gursoy@utu.fi (M.G.); 2 Global Health and Nutrition Science, IFF Health & Biosciences, Sokeritehtaantie, 20, 02460 Kantvik, Finland; johanna.hirvonen@iff.com (J.H.); arthur.ouwehand@iff.com (A.C.O.)

**Keywords:** 2′-fucosyllactose, 3-fucosyllactose, human beta defensins, cathelicidin, oral mucosal model

## Abstract

Human milk oligosaccharides (HMOs), the third largest solid fraction in human milk, can modulate inflammation through Toll-like receptor signaling, but little is known about their immunomodulatory potential in the oral cavity. In this study, we determined whether the HMOs 2′-fucosyllactose (2′-FL) and 3-fucosyllactose (3-FL) regulate human-beta defensin (hBD)-2 and -3, cathelicidin (hCAP18/LL-37), and cytokine responses in human gingival cells using a three-dimensional oral mucosal culture model. The model was incubated with 0.1% or 1% 2′-FL and 3-FL, alone and in combination, for 5 or 24 h, and hBD-2, hBD-3, and hCAP18/LL-37 were analyzed by immunohistochemistry. The expression profiles of interleukin (IL)-1, IL-1RA, IL-8, and monocyte chemoattractant protein (MCP)-1 were determined by LUMINEX immunoassay. The combination of 1% 2′-FL and 1% 3-FL, and 1% 3-FL alone, for 24 h upregulated hBD-2 protein expression significantly (*p* < 0.001 and *p* = 0.016, respectively). No changes in the other antimicrobial peptides or proinflammatory cytokines were observed. Thus, 3-FL, alone and in combination with 2′-FL, stimulates oral mucosal secretion of hBD-2, without effecting a proinflammatory response when studied in an oral mucosal culture model.

## 1. Introduction

Human milk contains an estimated 200 oligosaccharides (HMOs), which are composed of five building blocks with various bonds: glucose, galactose, N-acetylglucosamine, fucose, and N-acetylneuraminic acid [1,2]. The composition and concentrations of HMOs vary between mothers and over the lactation period [3,4,5]. Mature human milk contains 10–15 g/L oligosaccharides [6]. The HMOs 2′-fucosyllactose (2′-FL) and 3-fucosyllactose (3-FL) are trisaccharides, comprising lactose and an additional fucose; in 2′-FL, the fucose is attached to galactose, compared with glucose in 3-FL. The most abundant HMO in breast milk is 2′-FL, at approximately 2.7 g/L, and 3-FL is also present in relatively high concentrations, approximately 0.4 g/L [6]. HMOs serve as prebiotics, anti-adhesives, antimicrobials, and modulators of epithelial and immune cell function [7]. However, these effects are HMO structure-specific, and not all HMOs have the same effects [7].

In the oral cavity, the gingival epithelium acts as a barrier against constant bacterial stress. Epithelial keratinocytes secrete cytokines, chemokines, and antimicrobial peptides, all of which maintain a healthy homeostasis between the host and the oral microbiota. Human beta defensins (hBDs) and cathelicidin (hCAP18/LL-37) are cationic antimicrobial peptides with broad-spectrum antimicrobial activity. Expressed in clinically healthy gingival tissues, hBD 1-3 and hCAP18/LL-37 are upregulated during infection and inflammation [8]. Controlled stimulation of endogenous antimicrobial peptides is proposed as an alternative to antibiotic use in maintaining healthy host immune–bacterial interactions [9]. However, no such technique has been defined until now.

Immune responses in infants are underdeveloped, and human milk contributes directly to the infant’s antimicrobial defense [10]. Human milk components can modulate host pattern recognition receptors; for example, 2′-FL attenuates Toll-like receptor (TLR)-4 signaling, and 3′-galactosyllactose inhibits TLR-3 expression [11]. Based on findings that hBD-2 expression is regulated by TLR signaling [12], we hypothesized that HMOs may alter human gingival keratinocyte hBD and hCAP18/LL-37 expression. Thus, the aim of our study was to analyze the regulation of hBD, hCAP18/LL-37, and proinflammatory cytokine responses of two of the abundant HMOs, 2′-FL and 3-FL. We found that 3-FL induced hBD-2 protein expression in an organotypic oral mucosa model.

## 2. Results

### 2.1. Immunohistochemical Analysis of an Organotypic Oral Mucosa Model

**Figure 1:**
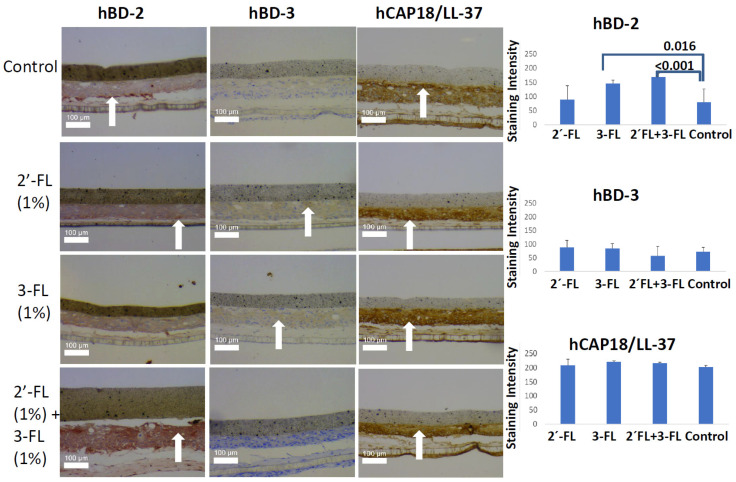
Human beta defensin 2 (hBD2) and 3 (hBD3) and cathelicidin (hCAP18/LL-37) protein levels in an oral mucosa model after incubation with 1% 2′-fucosyllactose (2′-FL), 3-fucosyllactose (3-FL), and their combination (2′-FL + 3-FL) for 24 h, and white arrows indicate regions of strong staining.

Based on our immunohistochemical analysis, 24 h incubation of an oral mucosa model with 1% 3-FL (*p* = 0.016) or 1% 2′-FL + 3-FL (*p* < 0.001) significantly upregulated hBD-2 (Figure 1). No other significant changes were observed compared with the control when 1% HMOs were incubated with the oral mucosa model for 5 h. Moreover, there were no significant differences on incubation with 0.1% HMOs for 24 h.

### 2.2. Proinflammatory Cytokine Analysis in Culture Medium

Interleukin (IL)-1β ranged between 0.4 and 1.2 pg/mL, leaving 34% of the samples under the limit of detection (Table 1). IL-1 receptor antagonist (IL-1RA) levels were 572-1959 pg/mL and did not differ either (Table 1). IL-8 levels were similar between treatment and control conditions at all time points, at 111233 and 299054 pg/mL (Table 1). Finally, monocyte chemoattractant protein (MCP)-1 levels ranged from 1570 to 2410 pg/mL, with no difference between treatment and control (Table 1).

## 3. Discussion

To the best of our knowledge, this is the first study to evaluate hBD-2, hBD-3, hCAP18/LL37, and proinflammatory cytokine responses in an oral mucosa model in response to two HMOs, 2′-FL and 3-FL. According to our results, 1% 3-FL, alone or in combination with 1% 2′-FL, upregulated the protein expression of hBD-2 secretion significantly. Because 2′-FL did not have any influence, it is likely that 3-FL drives the effect in the combination treatment and no antagonism with studied HMOs was observed.

HMOs are being increasingly incorporated into infant formulas, but their activation and inhibition of cellular responses have not been studied extensively. Based on our results, 3-FL, but not 2′-FL, induces hBD-2 protein expression. No other study has examined the interactions between 3-FL and hBDs in an oral mucosa model, preventing us from comparing our results with the literature. Kong et al. evaluated the effects of shear forces on the intestinal epithelial barrier with Caco-2 cells and, in addition, stimulated Caco-2 cells with 2′-FL, 3-FL, and lacto-N-triose II (LNT2) [13]. They found that shear force exposure downregulated antimicrobial peptide beta defensin (BD)-1 but that, under static and shear force conditions, LNT2—not 2′-FL or 3-FL—upregulated BD-1 [13]. However, with *Lactiplantibacillus plantarum* WCFS1, all three HMOs upregulated BD-1 under shear force conditions, and 2′-FL and LNT2 did so under static conditions [13].

Notably, milk hyaluronan also induces hBD-2 protein expression in colonic epithelial cells [14], indicating that various human milk proteins and other bioactive components may interact with the immune system during infant development [15,16]. The hBD-2-stimulating effect of 3-FL was observed only after the 24 h incubation. During infancy, the oral cavity is exposed to human breast milk frequently, often for several hours per day in total; feeding also occurs at night. HMOs are also being increasingly added to infant formula, and our results highlight the importance of evaluating the effects of individual HMOs on various outcomes outside of the gut. In human breast milk, the levels of HMOs vary between individuals and throughout lactation [3,4]. In this study, we used similar levels of 2′-FL and 3-FL to determine the effects of individual HMOs and detect possible synergies or antagonistic activity. Although 3-FL contains 2.5% residual lactose, the final concentration in the test medium (0.025%) seems too low to play any role in the observed effect as 80-fold higher concentrations were observed to be required for the induction of cathelicidin in vitro (after 48 h) [17].

Based on our results, cytokine secretion is not elicited by 2′-FL or 3-FL in the oral mucosal model. *In vitro* studies have demonstrated that 2′-FL suppresses the release of proinflammatory cytokines, reduces monocyte activation, and inhibits IL-8 expression during *Escherichia coli* infection [18,19,20]. We did not observe any immune-suppressive effect of 2′-FL, because our model did not include an immune stimulus—e.g., bacteria. However, it is important that 2′-FL and 3-FL do not induce inflammatory responses, which can be detrimental in the oral cavity.

A major strength of this study was its use of a three-dimensional (3D) oral mucosa model to track the cellular antimicrobial response against HMOs. An organotypic oral mucosal model contains gingival keratinocytes and gingival fibroblasts; thus, our model also included interactions between these two cell types. Moreover, this model can mimic underdeveloped oral mucosa better than adult oral mucosa as it lacks stippling, has a thinner epithelium, and has decreased density of connective tissue [21]. The advantage of a 3D model is that it is more sophisticated than a 2D monolayer model; however, in 2D monolayers, the cell numbers are close to same in all replicates. Long-term culture makes the 3D model sensitive to environmental conditions. In the present study, 0.1% 24 h, 1% 24 h, and 1% 5 h experiments were performed at independent time points. For this reason, we abstained from comparing the results between these groups, as cell numbers can differ between them. In the present model, the collagen layer, which carries the fibroblasts, was highly sensitive to physical trauma induced by sectioning. In the majority of the sections, as also visible in Figure 1, the collagen layer was damaged. For this reason, we limited our immunohistochemical analyses with the epithelium. Moreover, our model did not involve bacteria. Bacteria can modulate antimicrobial peptide gene expression and degrade antimicrobial peptides that are expressed by keratinocytes [22]. In vitro studies have shown that 2′-FL and 3-FL alter the adhesion and growth of various bacteria [23,24,25], necessitating an examination of the cellular responses that are induced by these HMOs in an organotypic mucosa–bacterial biofilm culture model. Application of different post-hoc tests led to different results in this study. For example, a *p* value of 0.023 was observed when IL-1RA concentrations were compared between 5 h control and 5 h 1% 2′-FL groups with LSD post-hoc test, while Tukey HSD gave a *p* value of 0.088. This was not surprising as LSD is less sensitive than Tukey HSD in post-hoc comparisons. However, this also indicates that there can be an effect of milk oligosaccharides on gingival cytokine secretion, which can be dependent on the selected model and experimental design.

In conclusion, HMO 3-FL induces hBD-2 in an organotypic oral mucosa model—an effect that increases in the presence of 2′-FL, suggesting that milk oligosaccharides may have cumulative effects on host cellular responses. These in vitro findings are encouraging but do require confirmation in the oral cavity in vivo.

## 4. Materials and Methods

### 4.1. 2′-Fucosyllactose and 3-Fucosyllactose Production and Purity

In this study, 2′-fucosyllactose (2′-FL) (CARE4U™ 2′FL, IFF Health & Biosciences, Kantvik, Finland) and 3-fucosyllactose (3-FL) (CARE4U™ 3FL, IFF Health & Biosciences, Brabrand, Denmark) were produced by commercial fermentation of primarily lactose and sucrose by genetically engineered *E. coli* K-12 strains (nonpathogenic and non-toxicogenic), purified, concentrated, and dried. The purity of the final 2′-FL product was ≥99%, and that of 3-FL product was 96% (2.5% lactose and 1.5% others) by high-performance liquid chromatography, expressed as area %.

### 4.2. Construction of Organotypic Oral Mucosal Model

A modified organotypic oral mucosa model was constructed, based on a reported method [26,27]. Briefly, gingival keratinocytes and gingival fibroblasts that were obtained from a healthy human gingival biopsy sample [28] were cultured at 37 °C in 5% CO_2_. The gingival fibroblasts were suspended at a density of 3 × 10^5^/mL in collagen solution (Vitrogen, Cohesion technologies, Palo Alto, CA, USA) and plated in 10 mm culture inserts (ThinCert, Greiner Bio-One, Monroe, LA, USA). Then, the inserts were placed in 12-well tissue culture plates for 24 h to solidify. The gingival keratinocytes were seeded on collagen–fibroblast gels at a density of 8 × 10^5^/mL, and when the cells reached confluence, the inserts were placed on metal grids to maintain the air–liquid interface. Then, the model was allowed to grow for an additional 14 days. The culture medium was formed of antibiotic-free Green medium consisting of Dulbecco’s modified eagle’s medium and Ham F-12 medium (3:1), 10% fetal calf serum, 5 ng/mL epidermal growth factor, 4 mM L-glutamine, 5 μg/mL insulin, 0.4 μg/mL hydrocortisone, 0.1 nM cholera toxin, 18 mM adenine, and 100 μg/mL ascorbic acid.

### 4.3. Stimulation of Organotypic Oral Mucosal Model and Sample Collection

Precut sterile nitrocellulose membranes were placed on the organotypic oral mucosa model. Then, 10 µl of 2′-FL, 3-FL, or their combination (2′-FL + 3-FL) was applied on top of the membranes. Each oligosaccharide was tested at 2 concentrations (0.1% and 1% *w*/*v*) for 2 incubation periods (5 and 24 h). For the control model, a “carrier” (distilled water) was added to the top of the membranes. After the incubations, the inserts were removed from the 12-well plates, and the organotypic oral mucosa model was fixed in 10% formalin for 16 h. The medium from each well was collected and stored at −80°C for cytokine analysis. The formalin-fixed model was dehydrated through a series of 70%, 96%, and 100% ethanol. After brief immersion in xylene, all samples were cut in half and placed in paraffin blocks, sheared side down. All experiments were performed in triplicate.

### 4.4. Immunohistochemical Examination of Organotypic Oral Mucosal Model

Paraffin-embedded samples of the organotypic oral mucosa model were cut into 5-μm-thick sections and placed on slides for immunohistochemical analysis. The sections were deparaffinized and immunostained for hBD-2, hBD-3, and hCAP18/LL-37 using routine procedures on an automated immunostainer (TechMate, DAKO, Glostrup, Denmark). Briefly, the sections underwent antigen retrieval in 1 mmol/L citrate buffer (pH 6.0) in a microwave twice for 5 min and incubated with 3% H_2_O_2_ to block endogenous peroxidase activity The primary antibodies—anti-human beta-defensin 2 (R&D Systems, AF2758, Minneapolis, MN, USA), anti-beta-defensin 3 (LifeSpan BioSciences, Inc., LS-B86, Seattle, WA, USA), and anti-human LL-37/CAP18 (Hycult Biotech, HM2070, Uden, The Netherlands)—were detected with biotinylated secondary antibody (Histofine Simple Stain MAX PO Universal Immuno-peroxidase Polymer Anti-goat, Nichirei Biosciences Inc., 414161F, Tokyo, Japan) and streptavidin–horseradish peroxidase and visualized with 3,3′diaminobenzidine tetrahydrochloride in horseradish peroxidase buffer.

The immunohistochemical stainings were evaluated under a light microscope (Leica DMLB, Leica, Wetzlar, Germany), and high-resolution images were captured to analyze signal intensities (Leica DC 300 V 2.0 Leica, Wetzlar, Germany) in ImageJ (version 1.46c; Rasband WS, National Institutes of Health, Bethesda, MD, USA) with the immunohistochemistry image analysis toolbox plugin, version 2 (National Institutes of Health, Bethesda, MD, USA).

### 4.5. Proinflammatory Cytokine Analysis

Levels of IL-1β, IL-1 Ra, IL-8, and MCP-1 in culture media were measured in triplicate by Luminex^®^ xMAP™ technique (Luminex Corporation, Austin, TX, USA) using commercially available kits (Human Cytokine, Chemokine, Growth Factor, and Diabetes Assays on Magnetic Beads, Human Group 1) without any dilution.

### 4.6. Statistical Analysis

The statistical analysis was performed by one-way ANOVA with Tukey’s HSD using SPSS V24.0 (IBM, Armonk, North Castle, NY, USA). The detection limit of the assay was 0.6 pg/mL for IL-1β, 1.0 pg/mL for IL-8, 5.5 pg/mL for IL-1Ra, and 1.1 pg/mL for MCP-1. *p* values less than 0.05 were considered to be statistically significant.

## Figures and Tables

**Table 1:** Proinflammatory cytokine levels (mean ± standard deviation) in oral mucosa model after incubation with 1% or 0.1% 2′-fucosyllactose (2′-FL) and 3-fucosyllactose (3-FL) and their combination (2′-FL + 3-FL) for 5 and 24 h.

	IL-1β (pg/mL)	IL-1RA (pg/mL)	IL-8 (pg/mL)	MCP-1 (pg/mL)
5 h				
Control	0.9 ± 0.5	1688 ± 847	2,70,293 ± 120527	2218 ± 303
1% 2′-FL	0.8 ± 0.3	619 ± 24	1,76,987 ± 83948	2253 ± 359
1% 3-FL	1.2 ± 0.3	1830 ± 161	2,99,054 ± 234387	2410 ± 618
1% 2′-FL+3-FL	0.8 ± 0.2	848 ± 361	2,05,258 ± 94,475	1911 ± 260
24 h				
Control	0.7 ± 0.2	1959 ± 167	1,14,840 ± 58,017	1832 ± 232
1% 2′-FL	0.6 ± 0.4	1736 ± 435	1,11,945 ± 94,296	1763 ± 371
1% 3-FL	0.9 ± 0.5	1877 ± 246	1,11,233 ± 37,557	1693 ± 74
1% 2′-FL+3-FL	0.5 ± 0.3	1698 ± 113	1,37,496 ± 78,958	1758 ± 229
24 h				
Control	0.4 ± 0.1	572 ± 94	70,376 ± 15,278	1570 ± 88
0.1% 2′-FL	1.2 ± 0.1	1542 ± 183	1,11,861 ± 36,202	1940 ± 504
0.1% 3-FL	1.1 ± 0.7	1206 ± 1035	15,9765 ± 1,23,521	1759 ± 244
0.1% 2′-FL+3-FL	1.0 ± 0.5	837 ± 618	1,46,936 ± 81,918	1782 ± 227

IL-1β = interleukin 1β; IL-1RA = interleukin-1 receptor antagonist; IL-8 = interleukin 8; MCP-1 = monocyte chemoattractant protein 1.

## Data Availability

Data available on request.
